# The variation in urinary calcium levels in adult patients with fracture and surgical intervention

**DOI:** 10.1186/s13018-017-0624-x

**Published:** 2017-08-15

**Authors:** Junfei Wang, Xin Zheng, Liming Zhang, Yifan Zhang, Jin Xiong, Yixin Cheng, Hongfei Shi, Xusheng Qiu, Leqin Zhou, Xizhao Sun

**Affiliations:** 1 Department of Orthopedics, Nanjing Drum Tower Hospital, the affiliated hospital of Nanjing University Medical School, 321 Zhongshan Road, Gulou District, Nanjing, Jiangsu Province 210008 China; 2 Department of Orthopedics, The Affiliated Hospital of Xuzhou Medical University, Xuzhou, 221006 China; 3 Department of Urology, Nanjing Drum Tower Hospital, the affiliated hospital of Nanjing University Medical School, Nanjing, 210008 China

**Keywords:** Hypercalciuria, Fracture type, Fracture location, Age, Gender

## Abstract

**Background:**

Generally, a higher calcium diet is fed to fracture patients after surgery. However, recent studies have indicated that higher dietary calcium intakes increase the risk of urinary stones for fracture patients. Therefore, this study aimed to observe the variation in urinary calcium levels among fracture patients who underwent surgery, based on fracture type, fracture location, age and gender.

**Methods:**

A total of 768 subjects were enrolled in this study from 2012 to 2015 and were divided into 2 groups: group A (fracture patients who underwent surgery) and group B (normal patients without fracture). Urine samples were collected for a 24-h period (24-h urine), at multiple specific time points before and after surgery for group A, or after hospitalisation for group B. Subsequently, urine calcium was detected and the changes were evaluated according to fracture location, fracture type, age and gender, as well as the distribution of hypercalciuria.

**Results:**

Compared with group B, the level of urine calcium in group A significantly increased at different time points during the study period (*P* < 0.05). There were significant differences in the changes in urine calcium levels according to fracture location, fracture type and age, but not gender. Further, there were more patients with hypercalciuria in group A at the different time points, compared with group B.

**Conclusion:**

Variation in urinary calcium among fracture patients that underwent surgery was of a regular pattern and hypercalciuria was also found in these patients. Therefore, a high-calcium diet and calcium supplements should be used with caution in this patient population.

## Background

Bone fracture is damage in the continuity of the bone [1]. Calcium and phosphate are part of the chemical composition of the bone [2], and calcium makes up about 1.5–2% of body weight, while approximately 99% of that calcium is present in the bones and teeth [3]. Dietary sources, vitamin D and calcium supplements can increase calcium intake to promote bone growth in children and prevent osteoporosis in elderly populations [4]. Additionally, a previous study showed that higher dietary calcium intake reduced the risk of fracture in older men and women [5]. However, other studies indicated that higher dietary calcium intakes increase the risk of urinary stones (2–13%) in fracture patients or long-term bedridden patients, compared with healthy populations [6–8]. In other words, the consequences of increased calcium intakes for patients with a fracture are uncertain. Normally, urinary stones result from hypercalciuria (when calcium levels in urine are more than 7.5 mmol/24 h in men and more than 6.25 mmol/24 h in women), which is the most common metabolic problem [9, 10]. Hypercalciuria also causes other complications, such as haematuria, frequency and urgency of urination and a weak urinary stream [9, 11, 12]. Therefore, it is important to study the variation in urinary calcium levels in fracture patients to guide pharmaceutical intervention and diet after surgery.

In the present study, we observed the variation in urinary calcium levels in fracture patients that underwent surgery. This was based on fracture type, fracture location, age and gender, as well as the distribution of hypercalciuria at different time points, in order to offer guidelines for the use of calcium supplements or diets in fracture patients after surgery.

## Methods

### Subjects

A total of 768 subjects (female = 271, male = 497, mean age = 46.69 ± 12.91, 18–97 years old) were enrolled in this study from 2012 to 2015. They were divided into two groups: group A and group B. Group A: 721 fracture patients (females = 309, males = 412, mean age = 46.60 ± 12.85 years) who underwent surgery within 1 week after fracture. Exclusion criteria were as follows: (1) patients who underwent fracture fixation through an emergency operation; (2) patients who were not admitted to hospital on the day of fracture; (3) patients in which an operation was not performed within a week after fracture; (4) patients who had multiple fractures, such as spinal fracture, rib fracture, pathological fracture, old fractures and delayed fracture healing or non-union; (5) patients who had any kind of fracture in the previous 3 months; (6) patients with a history of urinary calculus, parathyroid disease or renal chronic insufficiency. All the patients had surgery performed between 72 h and 7 days after fracture. Group B: 47 patients (females = 18, males = 29, mean age = 48.4 ± 13.98 years) without fracture in the present study, but who had undergone surgery for internal fixation in the previous 12 months. Exclusion criteria were as follows: (1) patients who had any kind of fracture in the last 3 months; (2) patients with a history of urinary calculus, parathyroid disease or renal chronic insufficiency. This study was approved by the ethics committee of Nanjing Drum Tower Hospital. The recruited subjects gave informed consent before commencing the present study.

### Study protocol

For each patient, urine was collected over a 24-h period (24-h urine), using a jug that contained preservative (2 mL methylbenzene) to prevent bacterial growth. The initial urine was removed at 7:00 am, and the 24-h urine was collected in the jug until 7:00 am the next day. The 24-h urine samples were collected on the first day (d), third day, first week (week; first day after operation), third week, fourth week, sixth week, eighth week and twelfth week from fracture patients in group A. For patients in group B, 24-h urine samples were collected on the first day, third day, first week, third week, fourth week, sixth week, eighth week and twelfth week after hospitalisation. Subsequently, the urine calcium in the two groups was detected using a Vitros 350 autoanalyzer (Johnson-Johnson, NY, USA). The changes in urine calcium levels were evaluated according to fracture location, fracture type, age and gender. Additionally, the distribution of hypercalciuria in groups A and B at the different time points was also analysed.

### Statistical analysis

The Wilcoxon unpaired two-sample test was used to compare the mean value of 24-h urine calcium levels between the different groups. The repeated measures data of analysis of variance (ANOVA) was used to analyse the changes in urine calcium levels at different time points. The least significant difference (LSD) test was used to analyse the differences among all groups. All data (mean ± SD) were analysed by using the Statistical Package for Social Sciences (SPSS) software (version 19.0, Chicago, IL, USA). *P < 0.05 was considered statistically significant.

## Results

### Changes in urine calcium levels at different time points

**Fig. 1:**
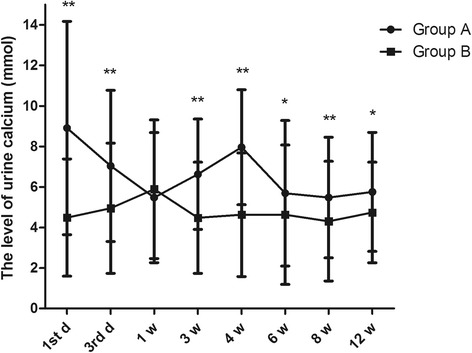
The changes of 24-h urine calcium at different time points in groups A and B. *P < 0.05, **P < 0.01

**Fig. 2:**
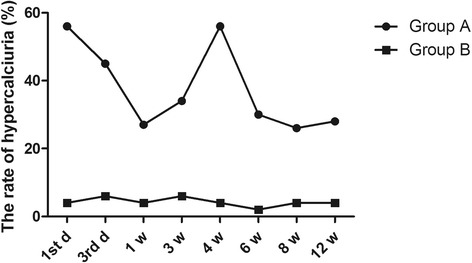
The distribution of hypercalciuria in groups A and B at different time points. *P < 0.05 and **P < 0.01 represent significant differences compared with group B

Compared with group B, the level of urine calcium in group A was significantly increased at different time points during the study period (*P* < 0.05), except at the first w (*P* = 0.397). Additionally, in group A, we found that the level of urine calcium was up to 8.91 ± 5.26 mmol on the first day (Fig. 1), which fell to 5.48 ± 3.21 mmol after 1 week, and increased again to 7.96 ± 2.84 mmol in the fourth week. It then reduced to normal levels in the sixth week and maintained homeostatic levels from the sixth week to the twelfth week. The two higher concentrations were more than 7.5 mmol, indicating hypercalciuria. However, the variation in urine calcium levels in group B was different. In group B, the level of urine calcium showed a tendency to rise from the first day to the first week (4.49 ± 2.89 to 5.89 ± 3.42 mmol). It then reduced to a homeostatic level from the fourth week, and all levels were less than 7.5 mmol. Moreover, the distribution of hypercalciuria in groups A and B was analysed at different time points. We found that there were more patients with hypercalciuria in group A at different times compared with that in group B (Fig. 2, *P* < 0.001.

### Changes in 24-h urine calcium levels according to fracture location

**Fig. 3:**
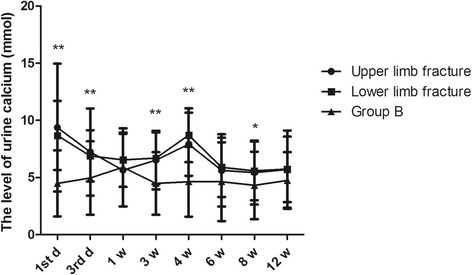
The effect of fracture location on the changes in 24-h urine calcium. Group A was divided into upper limb fracture and lower limb fracture groups, according to fracture location. *P < 0.05 and **P < 0.01 represent significant differences compared with group B

According to fracture location, all patients were divided into an upper limb fracture group (*n* = 202) and a lower limb fracture group (*n* = 519). The variation in urine calcium levels was similar between the upper limb fracture group and the lower limb fracture group. The level of urine calcium was up to 9.36 ± 5.59 mmol in the upper limb fracture group and 8.68 ± 3.01 mmol in lower limb fracture group on the first day (Fig. 3, *P* = 0.000), both fell to 5.64 ± 3.17 mmol and 6.55 ± 2.38 mmol after 1 week (*P* = 0.507), and again increased to 7.91 ± 2.77 mmol and 8.7 ± 2.36 mmol in the fourth week (*P* = 0.000), respectively. They both reduced to normal levels in the sixth week and maintained homeostatic levels from the sixth week to the twelfth week. The two higher concentrations in each group were both more than 7.5 mmol.

### Changes of 24-h urine calcium levels in different fracture types

**Fig. 4:**
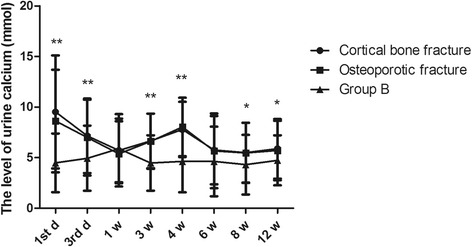
The effect of fracture type on the changes in 24-h urine calcium. Group A was divided into cortical fracture and osteoporotic fracture groups according to fracture type. *P < 0.05 and **P < 0.01 represent significant differences compared with group B

Based on the different fracture types, patients were divided into a cortical bone fracture group (*n* = 233) and an osteoporotic fracture group (*n* = 488). The variation in urine calcium levels was similar between the two groups. The level of urine calcium was up to 9.52 ± 5.59 mmol in the cortical bone fracture group and 8.62 ± 5.07 mmol in the osteoporotic fracture group on the first day (Fig. 4, *P* = 0.000). They both fell to 5.72 ± 3.14 mmol and 5.38 ± 3.23 mmol after 1 week (*P* = 0.281) and again increased to 7.79 ± 2.72 mmol and 8.04 ± 2.89 mmol in the fourth week (*P* = 0.000), respectively. They then reduced to normal levels in the sixth week and maintained homeostatic levels from the sixth week to the twelfth week. The two higher concentrations in the osteoporotic fracture group were more than 7.5 mmol, while only the concentration of urine calcium on the first day was more than 7.5 mmol in the cortical bone fracture group.

### Changes in 24-h urine calcium levels at different ages

**Fig. 5:**
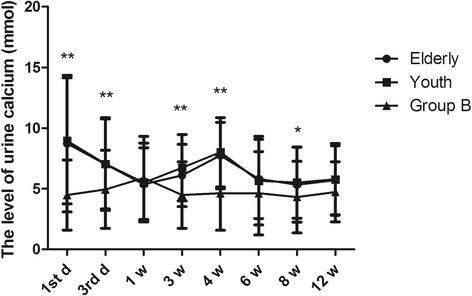
The effect of age on the changes in 24-h urine calcium in fracture patients. Group A was divided into elderly and young groups, according to age. *P < 0.05 and **P < 0.01 represent significant differences compared with group B

Based on their ages, all patients were divided into a young group (age < 60, *n* = 605) and an elderly group (age > 60, *n* = 116). The variation in urine calcium levels was similar between the two groups. The level of urine calcium was up to 8.72 ± 5.63 mmol in the elderly group and 8.95 ± 5.19 mmol in young group on the first day (Fig. 5, *P* = 0.000). They both fell to 5.37 ± 3.00 mmol and 5.51 ± 3.25 mmol after 1 week (*P* = 0.635) and again increased to 7.74 ± 2.74 mmol and 8.00 ± 2.85 mmol in the fourth week (*P* = 0.000), respectively. They then reduced to normal levels in the sixth week and maintained homeostatic levels from the sixth week to the twelfth week. The two higher concentrations in the young group were more than 7.5 mmol, while in the elderly group only the concentration on the first day was more than 7.5 mmol.

### Changes in 24-h urine calcium levels between males and females

**Fig. 6:**
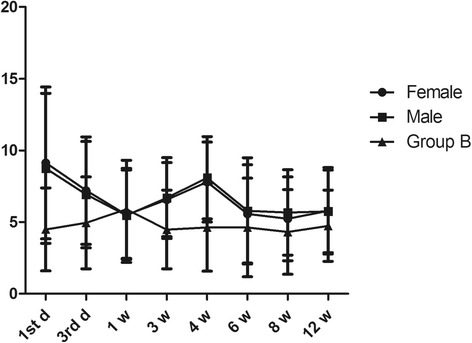
The effect of gender on the changes in 24-h urine calcium in fracture patients. Group A was divided into female and male groups according to gender. *P < 0.05 and **P < 0.01 represent significant differences compared with group B

All patients were divided into a female group (*n* = 309) and a male group (*n* = 412), according to gender. The variation in urine calcium levels was similar between the two groups (Fig. 6). The two higher concentrations at the first day and the fourth week in males were more than 7.5 mmol, while only the concentration of urine calcium on the first day was more than 7.5 mmol in females. There was no significant difference between the female group and male group.

## Discussion

In the present study, the level of urine calcium in group A significantly increased at different time points during the study period, compared with group B. Moreover, there were more patients with hypercalciuria in group A at different times. The variation in urinary calcium levels among fracture patients was of a regular pattern. In group A, the level of urine calcium increased on the first day, fell after 1 week, and again increased in the fourth week. It reduced to normal levels in the sixth week, which were maintained for the remainder of the study period. In addition, there were significant differences in the changes in urine calcium levels based on fracture location, fracture type and age but not gender.

As early as 1958, Hodkinson et al. considered urine calcium excretion of greater than 7.5 mmol/24 h in men or greater than 6.25 mmol/24 h in women to be hypercalciuria, and this is the gold standard for diagnosing hypercalciuria [13]. Subsequently, some studies have found that the random urinary calcium/creatinine (Ca/Cr) ratio is greater than 0.21 mg/mg in patients with hypercalciuria [14, 15]. There are many studies that have explored the correlation between Ca/Cr ratio and 24-h urinary calcium excretion [16, 17]. However, it is still controversial to use the Ca/Cr ratio instead of 24-h urine calcium excretion for diagnosis. Liu et al. indicates that the urinary Ca/Cr ratio cannot replace 24-h urine calcium excretion to diagnose hypercalciuria [18], whereas Rull et al. state that the Ca/Cr ratio is a simple predictive index of urine calcium for postmenopausal women with osteoporotic fractures [19]. Therefore, the method of 24-h urine calcium excretion, with >7.5 mmol/24 h defined as hypercalciuria, was used to evaluate the variation in urinary calcium for fracture patients in the present study.

In a previous study, the content of urine calcium increased in patients with pelvic fracture that underwent skin traction within 3 weeks of fracture [20]. Yusuf et al. showed that the level of calcium in blood and urine of trauma patients is significantly greater than baseline [6]. Similarly, the level of urine calcium in group A significantly increased at different time points during this study period, compared with group B. As reported, there is a higher incidence of hypercalciuria in postmenopausal women with osteoporotic fracture [19]. Meanwhile, there were more fracture patients with hypercalciuria after surgery in the present study. Therefore, fractures or wounds might induce a negative balance for calcium content in the body by increasing calcium excretion into urine. Additionally, calcium supplementation can increase urine calcium levels and can induce renal stone risk in healthy postmenopausal women [21]. Thus, wounds or calcium supplementation might be major risk factors for urinary stones in patients with fracture.

Hardy et al. found that the mean calcium level fell to 2.1 mmol/L (the normal range is from 2.1 to 2.6 mmol/L) within 24 h after fracture and then rose to a peak at 4 weeks [22]. Interestingly, the level of urine calcium in our fracture patients increased on the first day (fracture time), fell after 1 week (surgery time), then again increased after surgery in the fourth week. As for the above discussion, fractures or wounds might induce a negative balance of calcium content in the body by increasing calcium excretion into urine. In the fracture healing period, the calcium levels in the body are maintained at homeostatic levels through modulating osteoblasts and osteoclasts [23]. Type I collagen, the main component of bone matrix, is synthesised by Runx2 during early osteoblast differentiation [24]. At the surface of the bone, osteoclasts dissolve bone minerals to release calcium and degrade collagen fibres by enzymes and acids [25]. Therefore, such mechanisms may explain why urine calcium reduced to normal levels in the sixth week, and then homeostasis was maintained, in fracture patients after surgery.

Moreover, there was a significant difference in the changes in urine calcium levels based on fracture location, fracture type and age, but not gender, in the present study. In a previous study, immobilised trauma patients had higher calcium content in serum during the 4 weeks under study, compared to ambulant trauma patients [6]. In this study, upper limb fracture patients seemed to be like immobilised trauma patients, and lower limb fracture patients seemed to be like ambulant trauma patients. As a result, calcium levels were higher in the upper limb fracture patients than the lower limb fracture patients. Osteoporotic fracture occurs frequently in the older population, whose bone mineral density (BMD) is obviously lower than in the young [26]. Letavernier et al. report that hypercalciuria increased the risk of low BMD compared with controls, and showed a negative relationship with BMD [27]. It was the same in our results. The relationship between low BMD and hypercalciuria is that lower serum calcium can stimulate the secretion of parathyroid hormones to release calcium [28]. There is no evidence for a difference between genders in the change in urine calcium levels during the fracture healing period. Therefore, gender is not a major factor of the variation in urinary calcium levels in fracture patients.

There were some limitations in this study, such as the short follow-up time, the limited cross-section of patients and being a single-centre study. However, our results demonstrated a variation in urinary calcium levels for fracture patients after surgery. This will be helpful to reduce the risk of urinary stones in patients with fracture. In the future, the relationship between urine calcium and fracture location, fracture type or age should be further studied.

## Conclusions

In conclusion, wounds or calcium supplementation might be major risk factors for urinary stones in patients with fracture, both of which increase the secretion of calcium into urine. Therefore, high-calcium diets and calcium supplements should be used with caution.

## Abbreviations

ANOVA: Analysis of variance
BMD: Bone mineral density
Ca/Cr: Calcium/creatinine
LSD: Least significant difference
SPSS: Statistical Package for Social Sciences
